# Enzymatic Degumming of Rice Bran Oil Using Different Commercial Phospholipases and Their Cocktails

**DOI:** 10.3390/life11111197

**Published:** 2021-11-06

**Authors:** Mayara S. Rodrigues, Rafaela M. Dos Passos, Paula V. de A. Pontes, Marcela C. Ferreira, Antonio J. A. Meirelles, Christian V. Stevens, Guilherme J. Maximo, Klicia A. Sampaio

**Affiliations:** 1 Department of Food Engineering, Faculty of Food Engineering, University of Campinas, Campinas 13083-862, Brazil; mayararodrigues4181@hotmail.com (M.S.R.); r211223@dac.unicamp.br (R.M.D.P.); paulapontes15@gmail.com (P.V.d.A.P.); tomze@fea.unicamp.br (A.J.A.M.); maximo@unicamp.br (G.J.M.); 2 Faculty of Technology, University of Campinas, Limeira 13484-332, Brazil; marcela@ft.unicamp.br; 3 Department of Green Chemistry and Technology, Faculty of Bioscience Engineering, Ghent University, 9000 Ghent, Belgium; Chris.Stevens@UGent.be

**Keywords:** vegetable oils, phospholipase, enzymatic degumming, phospholipids

## Abstract

Rice bran oil is a highly nutritious vegetable oil, as it is rich in tocols and γ-oryzanol. Degumming is the first step in the vegetable oil refining process, and its main objective is the removal of phospholipids or gums. In the present study, enzymatic degumming trials were performed on crude rice bran oil using the phospholipases PLA1, Purifine^®^ PLC, their mixture (PLA1/PLC), and a cocktail known as Purifine^®^ 3G. Enzymatic degumming applying 50 mg/kg of PLA1 for 120 min resulted in a residual phosphorus content of 10.4 mg/kg and an absolute free fatty acid increase of 0.30%. Enzymatic degumming applying 300 mg/kg of Purifine^®^ PLC for 120 min at 60 °C resulted in a residual phosphorus content of 67 mg/kg and an absolute diacylglycerol increase of 0.41%. The mixture of phospholipases and the cocktail presented approximately 5 mg/kg of residual phosphorus content after the reaction times. For all degumming processes, the preservation of minor components such as tocols and γ-oryzanol were observed. These results indicate that the use of enzyme mixtures or their cocktails to attain low phosphorus content and high diacylglycerol/free fatty acid conversion during enzymatic degumming is a viable alternative.

## 1. Introduction

Rice bran oil (RBO) is a type of cooking oil obtained from rice bran (*Oryza Sativa* L.) through appropriate technological procedures [1]. The oil contains in its composition saponifiable and unsaponifiable fractions. Moreover, from a nutritional perspective, RBO is exceptionally rich in minor components, such as tocotrienols, phytosterols, and γ-oryzanol, where, if combined, exert a wide spectrum of biological activities [2].

In order to reach the edible condition, crude oil obtained as a result of the extraction process must be submitted to a refining process, whose objective is to remove impurities and undesirable compounds [3]. Degumming is the first step in the refining of vegetable oils, and its main goal is the removal of phospholipids or gums. The main phospholipids present in rice oil are: phosphatidylcholine (PC), phosphatidylinositol (PI), phosphatidylethanolamine (PE), and phosphatidic acid (PA) [4].

Phospholipids are classified according to their degree of hydration (hydratable and non-hydratable). Hydratable phospholipids (HPL) become insoluble in oil in the presence of water and are easily separated by centrifugation. Most non-hydratable phospholipids (NHPL) are complexed with calcium (Ca), magnesium (Mg), and iron (Fe) salts, and to be removed, they need the addition of a chelating agent (citric acid or EDTA) to sequester metal ions, allowing their precipitation and separation by centrifugation [5,6].

Enzymatic degumming is a process for the removal of phospholipids from crude oil using phospholipases (enzymes). The phospholipases hydrolyze the ester bonds present on the phospholipid molecules, and diacylglycerols (DAGs) or free fatty acids (FFA) are released [7]. The most commonly used phospholipases are: phospholipase A1 and phospholipase A2 that remove the fatty acid from position 1 and 2 with respect to glycerol, and phospholipase C (PLC) that hydrolyzes the bond between the acylglycerol and the phosphate group to release diacylglycerols (DAG) [3,7,8]. Besides phosphorus removal, the use of phospholipases has the advantage of oil yield increase, effluent generation decrease, and production costs decrease [4,7].

A new enzyme cocktail has been developed, Purifine^®^ 3G; the mixture consists of Purifine^®^ PLC, PLA2, phosphatidylinositol-specific phospholipase C (PI-PLC) that acts on the phosphatidylcholine (PC), phosphatidylethanolamine (PE), and phosphatidylinositol (PI) [9]. The enzymatic cocktail has a high efficient conversion of phospholipids (PLs) into mostly diglycerides (DAGs), phosphates, some free fatty acids (FFAs), and some lysophospholipids (LPLs) [10]. Therefore, the cocktail produces an FFA and DAG increase and phosphorus decrease, which are advantages over using only a single enzyme.

The use of enzymatic degumming increases the oil yield and brings advantages to the oil industry. In addition, the association of enzymatic degumming with other processes, such as the production of biodiesel, allows for the use of unrefined oil, making processing cheaper [11]. Thus, it is necessary to study the best process parameters and oil types for expanding industrial application. Therefore, the main objective of the present study is to evaluate process parameters such as enzyme concentration and reaction time, applied to RBO using Lecitase Ultra (PLA1), Purifine^®^ PLC, and Purifine^®^ 3G. The experimental results were evaluated concerning the residual phosphorus content (P-content), the FFA increase, the DAG increase, and the content of tocols and γ-oryzanol of the degummed RBO.

## 2. Materials and Methods

### 2.1. Raw Material and Reagents

The crude rice bran oil was kindly donated by IRGOVEL (Pelotas-RS, Brazil). All chemicals used are either ultra-performance liquid chromatography (UPLC) or analytical grade. Sodium hydroxide (NaOH) and citric acid (CA) were purchased from Sigma Aldrich (São Paulo, Brazil). The diolein standard (purity ≥ 99%), the tetradecane, and the derivatizing agent (BSTFA) were purchased from Sigma Aldrich (São Paulo, Brazil).

### 2.2. Enzymes

The phospholipase C type Purifine^®^ PLC was donated by DSM Company (Delft, The Netherlands) with an activity of 22,000 PLCU/g. The cocktail Purifine^®^ 3G (PLC + PI-PLC + PLA2) was donated by DSM Food Specialties (Delft, The Netherlands) with an activity of 16,900 PLCU/g. The phospholipase A type Lecitase Ultra (PLA1) was donated by Novozymes (The Netherlands) with an activity of 10 KLU/g. 

### 2.3. Physicochemical Analysis

The free fatty acid (FFA) content was determined by titration according to the AOCS official method Ca 5a-40 [12] and was expressed as % by weight of oleic acid. The fatty acid profile of crude rice bran oil was analyzed by gas chromatography (GC), according to the AOCS official method Ce 1–62 [13]. Phosphorus content was measured by inductively coupled plasma (ICP) according to AOCS official method Ca 20–99 [14]. The pH was measured directly with a pH electrode in the gums fraction.

The acylglycerol composition was measured according to the AOCS official method Cd 11b-91 [15]. Approximately 0.05 g of the oil samples was dissolved in 100 µL of tetradecane and 300 µL of derivatizing agent (BSTFA). The mixture was heated at 70 °C for 20 min. Then, 50 µL of derivatized sample was transferred to vials and diluted with 1 mL of hexane and injected in a gas chromatography (Agilent Technologies 7890A, Santa Clara, CA, USA, using GC Agilent 7890A, with OnColumn injection and DB-5HT capillary column (15 m × 0.32 mm i.d. ×0.10 μm film thickness). The diacylglycerols were identified using a diolein standard.

### 2.4. Nuclear Magnetic Resonance (NMR) Analysis

The analysis of phospholipid composition was measured by Nuclear Magnetic Resonance (NMR) employing a Bruker Avance III 400 MHz automatic spectrometer. Triphenyl phosphate was used as internal standard [16]. 

### 2.5. Analysis of Minor Components

The γ-oryzanol content was determined according to the Codex Alimentarius methodology [1], which uses spectroscopy, in which n-heptane is used as a solvent. First, a scan of the γ-oryzanol in heptane solution was carried out over the entire range of the UV-visible spectrum to determine the wavelength at which maximum absorption occurs. A calibration curve was, then, constructed with solutions of known concentrations (0.030–0.20 mg/mL) of γ-oryzanol in heptane at the maximum absorption wavelength. The determination of γ-oryzanol in crude rice bran oil was carried out by weighing approximately 0.02 g of the sample in a 25 mL volumetric flask and diluting with heptane. Then, the solution was read with 315 nm absorbance. 

The determination of tocols content was carried out according to the methodology of Ansolin et al. [17]. The oil was diluted in isopropanol to a concentration of approximately 8000 µg.mL^−1^. The samples were filtered through hydrophobic PTFE filters and, then, followed for analysis. For liquid chromatography analysis, was utilized a Waters Chromatographic system composed of an ultra-performance liquid chromatography (UPLC) model Acquity, coupled with a mass spectrometer. The separation of the compounds was performed on a UPLC BEH C18 column (1.7 µm, 2.1 mm × 100 mm) operating at 30 °C. The injection volume was 5 µL. All samples were analyzed in triplicate.

### 2.6. Water Degumming (WDG)

Water degumming was performed in order to verify the best percentage of water to be added. For the performance of the process, the crude oil was initially heated at 80 °C and water percentages of 3, 5, 7, and 10% (*w*/*w*)—relative to the oil mass—were added, and the mixture was homogenized with mechanical stirring (350 rpm) for 15 min and, then, centrifuged (10,000 rpm/15 min) for the separation of degummed oil from gum.

### 2.7. Chemical Conditioning (CC)

The chemical conditioning aimed to adjust the pH value for maximal enzyme activity. In this case, crude RBO was heated to 80–85 °C, and citric acid was added as a 30% aqueous solution. The oil followed high shear mixing (1 min/16,000 rpm), and then, the mixture was stirred for 15 min/350 rpm. Then, a 14% NaOH aqueous solution was added and followed a stirring period (1 min/16,000 rpm). After, the gums and the oil were separated by centrifugation (15 min/1000 rpm), and both were sent for analysis.

### 2.8. Enzymatic Degumming Experiments (PLA1, Purifine^®^ PLC, Purifine^®^ 3G, and Combinations)

The enzymatic degumming experiments were performed with 400 g of crude RBO. The first step of the enzymatic degumming process using PLA1, Purifine^®^ PLC, and Purifine^®^ 3G was carried out similarly to the steps of the chemical conditioning (CC) in order to adjust the pH, however, without the centrifugation step. The oil was conditioned for 15 min at 80 °C with stirring at 350 rpm. After conditioning, the temperature of the oil mixture was reduced to 52–60 °C, depending on the type of the enzyme. Then, a certain amount of water (3%, relative to the weight of the oil) and a predefined amount of PLA1 (10–70 mg/kg), Purifine^®^ PLC (100–400 mg/kg), the combination PLA1/PLC-1G (50–300 mg/kg), or Purifine^®^ 3G (300 mg/kg) were added. For PLA1 and PLC experiments, first, the ideal concentrations of the enzymes were found, and then, the reaction time was analyzed. The mixture was homogenized under high shear (16,000 rpm) for 1 min to disperse the enzyme in the oil/water emulsion. After, the oil mixture was kept at the required temperature under stirring (350 rpm) for a period of time (0–120 min). The degumming reaction was stopped by heating the mixture for 15 min at 85 °C. Subsequently, the oil mixture underwent centrifugation (15 min/1000 rpm) to separate the degummed oil from the gums. 

### 2.9. Statistical Analysis

All measurements were performed in triplicate with all data expressed as mean value ± standard deviation of independent experiments in triplicate. Statistical analysis was performed with STATISTICA 7.0. The differences among the means were determined by the Tukey test. Significant differences were declared at *p* ≤ 0.05.

## 3. Results

The fatty acid composition, free fatty acid content, acylglycerol composition, and minor components such as tocols and γ-oryzanol content of crude rice bran oil are listed in Table 1. As expected, rice bran oil is mostly composed of TAG, but important amounts of acylglycerols were also detected. The free fatty acids, which are final degradation products of TAGs, represent about 5% of the crude oil. Rice bran oil contains oleic acid as the predominant fatty acid, followed by linoleic and palmitic acid, with minor amounts of additional fatty acids. Similar results were obtained by Lüdtke [18], where the levels of fatty acids in greater proportion in rice bran were: oleic (32.8–35.6%), linoleic (30.8–33.5%), and palmitic (19.5–21.1%).

According to the results, β/γ-tocotrienol presented the highest concentration in crude rice bran oil followed by β/γ-tocopherol and α-tocopherol. Tocopherols and tocotrienols are important antioxidants present in rice bran oil, which are related to the prevention of coronary disease, cataract formation, and lowering the levels of plasma triacylglycerols and cholesterol [19]. The γ-oryzanol content was analyzed, and the crude rice bran oil presented a content of 1.8%. Similar results were obtained by Van Hoed et al. [20] when evaluating the influence of chemical refining on the major and minor compounds of rice bran oil. According to Wang et al. [21], γ-oryzanol is efficient in serum normalization of total cholesterol, triglycerides, and LDL-cholesterol, and it induces FFA level reductions and high-density lipoprotein (HDL) cholesterol increases.

Table 2 shows the minerals content and the phospholipids composition present in crude rice bran oil. The total phosphorus content, determined by the inductively coupled plasma (ICP) at crude rice bran oil, is at a level of 426 mg/kg, while the phospholipids content evaluated by P-NMR reached 0.91%. The most relevant minerals for the oil refining process are P, Ca, Mg, and Fe. Residual phosphorus (>10 mg/kg) can cause oil darkening, resulting in off-flavors and imposing difficulties on the oil downstream processing [22]. In addition, the amounts of Ca, Fe, and Mg are directly related to non-hydratable phospholipids and, consequently, to the facility of the degumming process.

Rice bran oil was also analyzed concerning its phospholipid composition, and according to the results, phosphatidylcholine (PC), together with phosphatidylethanolamine (PE), represents 0.64% of the oil that contains a total of 0.91% of phospholipids. The oil also contains additional amounts of phosphatidylinositol (PI) and phosphatidic acid (PA), which can also be degraded by phospholipases. 

### 3.1. Water Degumming (WDG)

**Figure 1:**
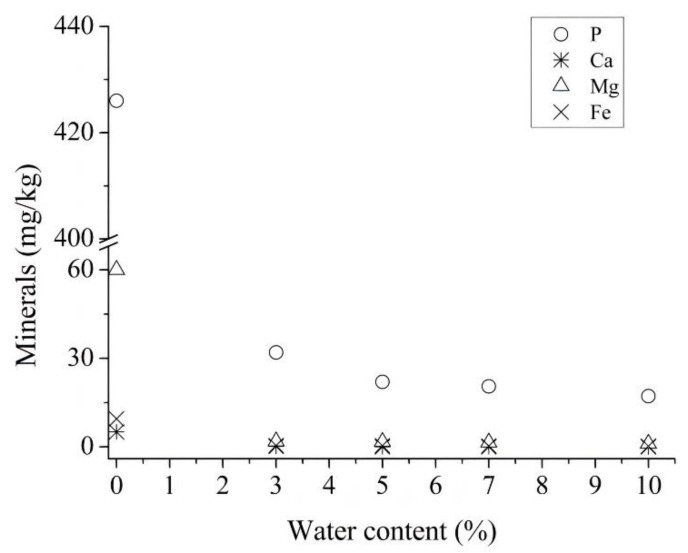
Minerals content after water degumming of crude rice bran oil.

The water degumming process has the main goal of removing the so-called hydratable phospholipids. The process was carried out using water proportions ranging from 3 to 10% *w*/*w*, relative to the oil mass. Figure 1 shows the minerals content found for the different proportions of water. According to Lamas, Constenla, and Raab [23], the phospholipids remaining after the water treatment can be considered as non-hydratable phospholipids (NHPL).

As can be seen, there was a reduction in the content of all minerals present in crude rice bran oil after water degumming. The phosphorus content in the crude oil was reduced from 426 mg/kg to 32.0, 22.0, 20.5, and 17.2 mg/kg when adding 3, 5, 7, and 10% water, respectively. However, there was only a small reduction in the phosphorus content (32.0–17.2 mg/kg) when the water content was increased from 3 to 10%, not showing great advantages for the process, besides increasing the costs of process and the consequent generation of effluents. Thus, 3% of water would already be a suitable value for carrying out the aqueous degumming process.

### 3.2. Enzymatic Degumming with PLA1

Enzymatic degumming is used for removing phospholipids or gums, and the process is carried out using phospholipases. The phospholipase A1 (PLA1) is used industrially, and due to its action in the sn1 position of the phospholipid, this enzyme produces free fatty acids and lysophospholipids. Fatty acids from the hydrolysis of phospholipids participate in increasing the oil yield, while lysophospholipids are removed by centrifugation, constituting the gums.

In general, a chemical conditioning using citric acid and sodium hydroxide is required to improve the hydratability of the non-hydratable phospholipids and to achieve the optimal pH value for the enzyme. When the chemicals were added to the vegetable oil, the content of phosphorus in the crude rice bran oil decreased from 426 mg/kg to 88 mg/kg. Similar results were obtained by Sampaio et al. [8] and Jiang et al. [24] when studying the degumming of crude corn and soybean oil.

**Figure 2:**
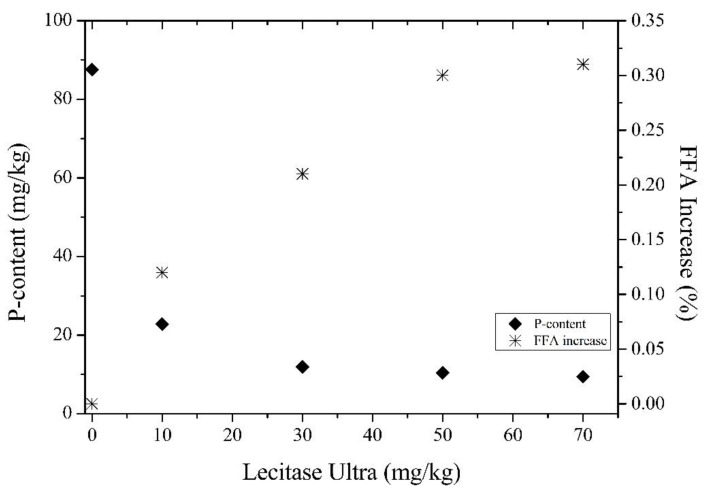
Effect of enzyme (PLA1) concentration on the residual phosphorus content and absolute FFA increase (reaction conditions: 120 min; 52 °C; 3% water; pH 5.1).

The PLA1 (Lecitase Ultra) experiments were performed with the addition of different enzyme concentrations to crude rice bran oil in order to monitor the changes in the residual phosphorus and FFA content. Figure 2 shows the phosphorus content and the FFA content as a function of the enzyme concentration within a reaction time of 120 min. As can be observed, the phosphorus content decreases, and the FFA content increases as the enzyme concentration increases. For enzyme concentrations varying from 10 to 50 mg/kg, the phosphorus content was reduced from 22.8 to 10.4 mg/kg, respectively. When the enzyme dosage was increased to 70 mg/kg, the residual phosphorus content was reduced only to 9.5 mg/kg. Regarding the FFA content, the evaluation of the FFA increase is also based on the amount of free fatty acids (4.97%) present in the degummed oil after the chemical conditioning. The enzyme Lecitase Ultra (PLA1) used in this work acts selectively in the sn1 position of the phospholipid, releasing fatty acids. Theoretically, conversion of 0.1% of phospholipids gives a formation of 0.036% FFA [25]. Therefore, as the crude rice bran oil has 0.91% of phospholipids, the generation of 0.33% of acidity would be expected. With an increase in the PLA1 dosage from 10 to 50 mg/kg, the absolute FFA increases linearly from 0.12% to 0.30%. However, a further increase in the enzyme concentration (70 mg/kg) results in a very poor FFA increase (0.31%), possibly due to the complete consumption of the substrate (gums). Considering these results, and the fact that 10 mg/kg is the maximum value to the degummed oil follow the physical refining [26], the enzyme dosage was set as standard at 50 mg/kg.

**Figure 3:**
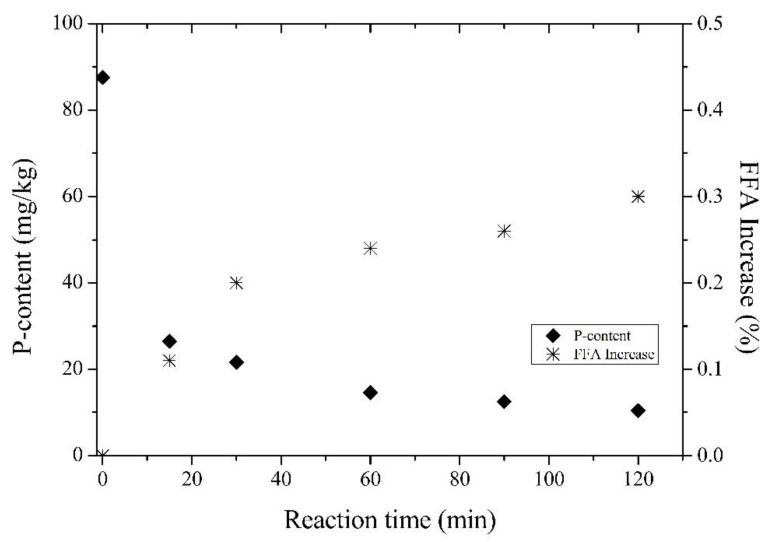
Effect of the enzyme (PLA1) reaction time on the residual phosphorus content and absolute FFA increase in the degummed oil (reaction conditions: 50 mg/kg; 52 °C; 3% water; pH 5.1).

Figure 3 shows the residual phosphorus content and the absolute FFA content obtained after the degumming process of crude rice bran oil with the enzyme Lecitase Ultra (PLA1) for different reaction times. By increasing the reaction time from 15 to 60 min, the content of residual phosphorus decreases from 21 to 15 mg/kg, while the absolute FFA content increases from 80.11% to 0.24%. A further increase in the reaction time from 90 to 120 min resulted in a phosphorus decrease from 13 to 10 mg/kg, and an absolute FFA increase varying from 0.26 to 0.30%. Therefore, 120 min of reaction time was fixed as an optimum value for the reaction time of crude rice bran oil.

Jahani et al. [27] studied the enzymatic degumming of rice bran oil using the enzyme Lecitase Ultra obtained from an experimental sample of Thermomyces lanuginosus/Fusarium oxysporum, with a concentration of 50 mg/kg. According to the authors, a reduction in the phosphorus content to values <10 mg/kg was achieved after a reaction time of 240 min. This result is probably due to the use of an older enzyme generation.

### 3.3. Enzymatic Degumming with Purifine PLC

**Figure 4:**
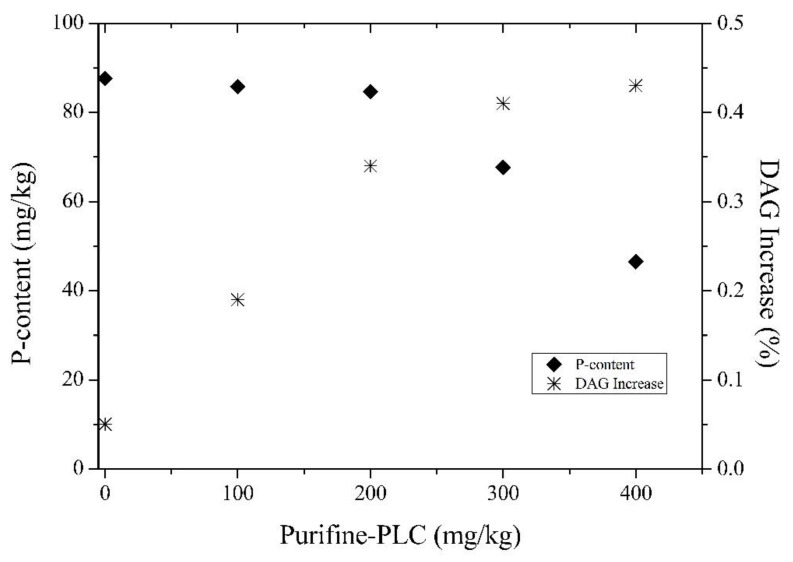
Effect of Purifine^®^ PLC dosage on the residual phosphorus content and absolute DAG increase (reaction conditions: 120 min; 60 °C; 3% water; pH 6.0).

The enzyme Purifine^®^ PLC is a phospholipase that acts selectively on the phospholipids PC and PE. The phospholipase C hydrolyzes the bond between the acylglycerol and the phosphate group realizing diacylglycerols (DAG) that are recognized as part of the oil, and hence contributing to the oil yield. Figure 4 shows the residual phosphorus content and the absolute DAG increase as a function of different Purifine PLC dosage. It is noticeable that for enzyme concentrations varying from 100 to 200 mg/kg, the residual phosphorus content remains practically constant. When enzyme concentrations varying from 300 to 400 mg/kg are added, the residual phosphorus content is reduced to values of 68 and 47 mg/kg, respectively. According to Gupta [9], depending on the degummed oil and the process employed, the P-content can reach values greater than 100 mg/kg.

Concerning the relative DAG increase, its evaluation was also carried out considering that the Purifine PLC enzyme acts specifically towards PC and PE, which results in a total of 0.65% (Table 2). According to Dayton and Galhardo [28], the amount of DAG that may be generated can be calculated using the original PC + PE content of the oil multiplied by the ratio between the molecular weight of the diolein and the average molecular weights of the phospholipids (605/750). An observed reaction efficiency of approximately 85% is used, and it is explained experimentally by the complete reaction of PC, but only partial reaction of PE, due to the relative rate of hydration. Thus, for a phospholipid content of 0.65%, a total DAG increase of 0.45% would be expected. The DAG content varied from 2.22% for the chemical conditioned oil, up to a level of 2.63% when using a concentration of 300 mg/kg of enzyme. Therefore, after degumming, the absolute DAG increase was 0.41%. Further increase in the enzyme concentration to 400 mg/kg resulted in only a 3% DAG increase. Considering the obtained results, it was established that 300 mg/kg is the most suitable enzyme concentration for the degumming of rice bran oil.

**Figure 5:**
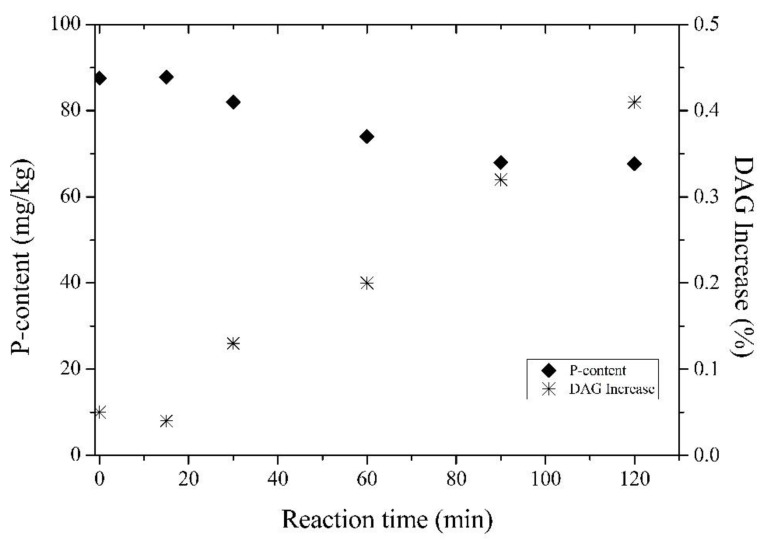
Effect of enzyme (PLC) reaction time on the residual phosphorus content and absolute DAG increase (reaction conditions: 300 mg/kg Purifine^®^ PLC; 60 °C; 3% water; pH 6.0).

After defining the best concentration of Purifine^®^ PLC to carry out the experiments applied to crude rice bran oil, the reaction kinetics was evaluated. Thus, enzymatic degumming was performed with Purifine^®^ PLC for a concentration of 300 mg/kg at different reaction times. Figure 5 shows the effect of the reaction time on the absolute DAG increase and residual phosphorus content of degummed rice bran oil. By adding 300 mg/kg of enzyme and increasing the reaction time from 15 to 60 min, it is possible to see that the residual phosphorus content decreased from 85 to 75 mg/kg, while the absolute DAG content increased from 0.04 to 0.20%. With an increase in the reaction time from 90 to 120 min, the residual phosphorus content remained practically constant (68–67 mg/kg), while the relative DAG increase varied from 0.32–0.41%. Hence, 120 min of reaction time was fixed as an optimum value for Purifine PLC reaction for crude rice bran oil.

Sampaio et al. [8] studied the enzymatic degumming of corn oil using 200 mg/kg of the Purifine^®^ PLC enzyme and found that, at 120 min of reaction time, the relative increase in the DAG content recached 0.54%, while the P-content was of 27 mg/kg. The authors also found that an increase in the reaction time to 240 min contributed only to a slight increase in DAG content and a decrease in the residual P, not justifying the adoption of a longer reaction time.

### 3.4. Enzymatic Degumming with Purifine^®^ 3G and Combinations (Purifine PLC/PLA1)

As Purifine PLC is an enzyme that acts selectively towards PC and PE, the obtained degummed oil has a phosphorus content comparable with water degummed oil, with phosphorus levels varying from 50 to 100 mg/kg. On the one hand, the enzyme Lecitase Ultra (PLA1) degrades all the phospholipids, but it produces FFA as a neutral oil, and higher FFA content in the degummed oil requires a larger fatty acid stripper, and it also promotes small additional oil loss when the FFA content increases. A possible solution to the mentioned issues can be the use of a mixture of PLC and PLA or other enzyme cocktails such as Purifine 3G added to the crude oil. Purifine^®^ 3G cocktail is a set of phospholipases that acts on all phospholipids (PA, PI, PC, and PE), as it consists of a mixture of the enzymes Purifine^®^ PLC, PLA2, and PI-PLC [9]. Therefore, it acts not only by increasing the oil yield (DAG and FFA), but also by reducing the minerals content. It is worth mentioning that within the literature, there are only few works on Purifine^®^ 3G enzyme until the present moment [10].

The addition of the enzyme mixture (PLC/PLA) and the enzyme cocktail (Purifine 3G) to crude rice bran oil was performed in both cases for two different reaction times, 60 min and 120 min. For the dosage of the enzyme mixture, we chose the optimum concentrations found previously (PLA1:50 mg/kg and PLC: 300 mg/kg), and in the case of the cocktail (Purifine 3G), the supplier data (300 mg/kg) were used. The obtained degummed oil was evaluated taking into account the DAG and FFA content and also the residual phosphorus content.

Table 3 shows the residual phosphorus content, the DAG and FFA content of the degummed rice bran oil after the action of the enzyme mixture, and the cocktail for reaction times of 60 min and 120 min. As can be seen, the enzyme mixture as well as the cocktail were effective in reducing the residual phosphorus content to values lower than 5 mg/kg for both reaction times. These results indicate that the use of either enzyme for 60 min of reaction time is already sufficient for the degumming of rice bran oil under the conditions employed. Regarding the oil yield increase, which was evaluated by the quantification of FFA and DAG content in the degummed rice bran oil, it can be seen that a longer reaction time resulted in practically constant values for the FFA content; however, it presented a higher DAG content, probably due to a high conversion extent. It is important to highlight that this behavior was observed for the enzyme mixture as well as for the cocktail.

Jiang et al. [4] studied enzymatic degumming on eight different varieties of vegetable oils using a mixture of enzymes PLC/PLA1 for 3.5 h of reaction time (0.5 h used for acid pretreatment and 3 h for enzymatic degumming), and the authors found a residual phosphorus content lower than 5 mg/kg for all oils, therefore, indicating the effectiveness of the use of the enzyme mixture added to crude vegetable oils.

### 3.5. Minor Components (Tocols and γ-Oryzanol)

The minor components present in rice bran oil, which correspond to the constituents of vitamin E (tocopherols and tocotrienols) and γ-oryzanol, constitute a great advantage to the vegetable oil and were evaluated after the degumming treatments. Table 4 presents the tocol content after water degumming and enzymatic degumming using PLA1 and Purifine^®^ PLC for different water concentrations and reaction times, respectively. As can be seen, among the tocols, β/γ-tocotrienol presented the highest value in the degummed oil, followed by β/γ-tocopherol, α-tocopherol, and α-tocotrienol. δ-tocopherol content was not detected in the oil. Similar results were found by Lüdtke [18] when studying the water degumming of crude rice bran oil.

Regarding the water degumming process, it is possible to verify that increasing the amount of water from 0 to 3% contributed to a significative reduction in tocol content when comparing to crude rice bran oil. The tocol losses reached approximately 9% for the highest amount of water added (10%), and these losses can be related to own process conditions such as high temperatures and oxidation reactions. When evaluating the enzymatic degumming processes using PLA1 and PLC for different reaction times, it can be observed that the chemical conditioning already promotes a reduction in tocol content, and it probably happens due to removal of part of the tocols during the degumming treatment that uses acids and base, besides the reaction time use for the process. Van Hoed et al. [29] verified that rice bran oil presented a relative change in the tocols’ composition when evaluating the physical refining deacidification, and the authors attribute the changes to the evaporation process caused by the temperatures and the low pressures.

Table 4 shows the minor components in the rice bran oil that underwent degumming with PLA1 within a reaction time of 120 min. The loss of tocol content was of approximately 10.5% from 0 to 120 min. Only the total tocol content of water degumming presented a significant difference from crude rice bran oil, and all enzymatic degumming did not differ significantly from chemical conditioning. Van Hoed et al. [29] utilized a physical deacidification per column for rice bran oil that consisted of an adapted type of washing and found that the rice bran oil presented a change in the relative composition of the tocols.

Table 5 shows the tocol content after the use of the enzyme mixture and the cocktail. According to the results, it can be seen that the β/γ-tocotrienol presented the highest values, thus, remaining in accordance with the results for the other types of degumming previously performed without a mixture. One should also point out that Purifine^®^ 3G cocktail showed significant β/γ-tocopherol differences for 60 and 120 min, and a similar difference was observed for β/γ-tocotrienol regarding the enzyme mixture, which can be related to losses associated to the processes.

The γ-oryzanol content represents an important compound present in rice bran oil, and it was measured in the present study after all the degumming processes. It was observed that the content of γ-oryzanol present in the oil after chemical conditioning was 1.7%, and the γ-oryzanol content remained in the range of 1.6–1.7% for all degumming processes, with no major changes. The present study is in accordance to Krishna [30] who was able to prove that the content of γ-oryzanol in the oil was not affected by degumming.

## 4. Conclusions

The present study showed that the use of PLA1, enzyme mixture (PLA1/Purifine PLC), and enzyme cocktail (Purifine 3G) was effective in reducing the residual phosphorus content of degummed rice bran oil to less than 10 mg/kg under the optimal conditions. However, rice bran oil degummed with Purifine PLC alone would require further treatment in order to be adequate for physical refining. After PLA1, about a 0.30% of absolute FFA increase was obtained. Degumming with PLC enzyme did not result in an FFA increase, but it increased the absolute DAG content (0.41%) by hydrolyzing PC ad PE. Treatments with enzyme mixtures (PLA1/PLC) and the cocktail (PLC 3G) resulted in both FFA and DAG increases in levels varying between 85 and 95% of the total phospholipid content. Concerning the minor components, which are represented in degummed rice bran oil by tocols and γ-oryzanol, it was verified that the chemical conditioning promoted a significant decrease in tocol content, while the γ-oryzanol content remained practically unchanged when comparing with the crude oil. For the enzyme treatments at different reaction times, there was a significant reduction when comparing the tocols results with chemical conditioning; however, when evaluating for the different reaction times, the tocols’ reduction was not always significant. Therefore, it can be concluded that enzymatic degumming with PLA1, Purifine^®^ PLC, enzyme mixture (PLA1/PLC), and the cocktail (Purifine^®^ 3G) is efficient in degumming rice bran oil and can be used industrially.

## Figures and Tables

**Table 1:** Characterization of crude rice bran oil.

**Parameter**	**RBO ^1^**
TAG (%)	92.73 ± 0.03
DAG (%)	2.17 ± 0.04
MAG (%)	0.13 ± 0.00
FFA (%)	4.97 ± 0.02
**Fatty Acid Composition**	**(%)**
Miristic (C14:0)	0.28 ± 0.02
Palmitic (C16:0)	20.20 ± 0.01
Estearic (C18:0)	1.75 ± 0.10
Oleic (C18:1)	39.56 ± 0.05
Linoleic (C18:2)	36.37 ± 0.80
Linolenic (C18:3)	1.84 ± 0.02
**Minor Components**	**(mg/kg)**
α-tocopherol	146.96 ± 0.12
β/γ-tocopherol	149.70 ± 0.28
α-tocotrienol	38.22 ± 0.54
β/γ-tocotrienol	461.45 ± 2.89
Total	796.33 ± 26.8
γ-oryzanol (%)	1.80 ± 0.03

^1^ RBO: rice bran oil. Content is the mean of triplicate ± S.D.

**Table 2:** Minerals content and phospholipids composition in the crude rice bran oil.

Parameter	RBO ^1^
**Minerals (mg/kg)**	
P	426.0 ± 6.0
Ca	9.60 ± 0.06
Fe	5.05 ± 0.08
Mg	60.0 ± 0.20
**Phospholipids (%)**	
PC	0.39 ± 0.04
PE	0.25 ± 0.02
PI	0.20 ± 0.01
PA	0.07 ± 0.03
Total (%)	0.91 ± 0.03

^1^ RBO: rice bran oil. Results of minerals content. Phospholipids composition. Contents are the mean of triplicate.

**Table 3:** DAG content, FFA content, and P-content of degummed rice bran oil for enzyme mixture and cocktail at different reaction times.

Combinations and Cocktail	Reaction Time (min)	DAG (%)	FFA (%)	P-Content (mg/kg)
CC	0	2.22 ± 0.19 ^a^	4.99 ± 0.14 ^a^	87.6 ± 0.04 ^a^
PLC-1G/PLA_1_	60	2.52 ± 0.02 ^a^	5.24 ± 0.02 ^a^	5.22 ± 0.02 ^b^
PLC-1G/PLA_1_	120	2.64 ± 0.06 ^a^	5.29 ± 0.00 ^a^	5.26 ± 0.01 ^b^
PLC-3G	60	2.62 ± 0.05 ^a^	5.21 ± 0.00 ^a^	5.00 ± 0.50 ^b^
PLC-3G	120	2.76 ± 0.15 ^a^	5.27 ± 0.04 ^a^	5.01 ± 0.12 ^b^

Chemical conditioning (CC). ^a, b^: Different letters in the same column indicate significant differences (*p* < 0.05).

**Table 4:** Content of tocols after water degumming and enzymatic degumming using PLA_1_ and Purifine^®^ PLC.

**Water Content (%)**	**β/γ-Tocotrienol**	**α-Tocotrienol**	**β/γ-Tocopherol**	**α-Tocopherol**	**Total (mg/kg)**
0^*^	461.45 ± 2.89 ^a^	38.22 ± 0.54 ^a^	149.70 ± 0.28 ^a^	146.96 ± 0.12 ^a^	796.33 ± 18.13 ^a^
3	460.11 ± 2.80 ^a^	19.22 ± 2.80 ^b^	120.21 ± 1.81 ^b,c^	133.23 ± 1.10 ^a^	732.77 ± 1.84 ^b^
					
					
10	438.46 ± 2.92 ^a,b^	15.02 ± 1.16 ^b^	116.15 ± 2.12 ^c^	124.00 ± 2.30 ^b^	693.63 ± 15.3 ^b^
**PLA_1_ (min)**	**β/γ-Tocotrienol**	**α-Tocotrienol**	**β/γ** **-Tocoferol**	**α-Tocopherol**	**Total (mg/kg)**
0	458.30 ± 1.26 ^a^	29.18 ± 0.02 ^a,b^	136.11 ± 0.01 ^b^	140.23 ± 0.04 ^a^	763.82 ± 21.16 ^a,b^
					
30	408.70 ± 2.13 ^b,c^	17.54 ± 0.62 ^b^	148.06 ± 0.83 ^a^	145.28 ± 1.28 ^a,b^	719.58 ± 14.3 ^b^
60	390.56 ± 3.80 ^b,c^	16.54 ± 0.23 ^b^	152.56 ± 1.24 ^a^	143.43 ± 0.45 ^a,b^	703.09 ± 2.64 ^b^
					
120	408.42 ± 0.31 ^b,c^	16.02 ± 0.07 ^b^	137.53 ± 0.45 ^a^	121.62 ± 0.86 ^b^	683.59 ± 11.1 ^b^
**Purifine PLC (min)**	**β/γ-Tocotrienol**	**α-Tocotrienol**	**β/γ-Tocopherol**	**α-Tocopherol**	**Total (mg/kg)**
0	457.6 ± 1.20 ^a^	28.17 ± 0.02 ^a,b^	136.15 ± 0.01 ^b^	141.23 ± 0.03 ^a,b^	763.15 ± 20.10 ^a,b^
					
30	408.70 ± 5.72 ^b,c^	17.54 ± 0.43 ^b^	148.06 ± 0.76 ^a^	145.28 ± 1.54 ^a,b^	719.58 ± 6.77 ^b^
60	409.31 ± 3.40 ^b,c^	17.12 ± 0.30 ^b^	146.83 ± 3.05 ^a^	144.15 ± 2.36 ^a,b^	717.41 ± 5.24 ^b^
					
120	420.69 ± 1.15 ^a,b^	20.00 ± 0.16 ^b^	124.42 ± 0.59 ^b,c^	131.31 ± 0.48 ^b^	696.42 ± 9.60 ^b^

Crude rice bran oil (0*); chemical conditioning (0). ^a, b, c^: Different letters in the same column indicate significant differences (*p* < 0.05).

**Table 5:** Tocol content after enzymatic degumming using combination (PLCPLA_1_) and cocktail (Purifine^®^ 3G).

Treatments	Time (min)	β/γ-Tocotrienol (mg/kg)	α-Tocotrienol (mg/kg)	β/γ-Tocopherol (mg/kg)	α-Tocopherol (mg/kg)	Total (mg/kg)
CC	0	458.30 ± 1.30 ^a^	29.20 ± 0.50 ^a^	136.10 ± 0.34 ^a^	140.20 ± 0.28 ^a^	763.8 ± 21.2 ^a^
PLC/PLA1	60	436.76 ± 0.35 ^a^	29.40 ± 0.70 ^a^	151.85 ± 0.22 ^a^	137.05 ± 0.23 ^a^	755.06 ± 9.89 ^a^
PLC/PLA1	120	404.18 ± 1.24 ^b^	27.45 ± 0.80 ^a^	152.86 ± 0.21 ^a^	135.81 ± 0.30 ^a^	720.30 ± 14.71 ^a^
PLC-3G	60	452.14 ± 1.60 ^a^	22.83 ± 1.30 ^a^	129.88 ± 0.44 ^b^	138.21 ± 0.38 ^a^	743.06 ± 1.41 ^a^
PLC-3G	120	433.47 ± 1.40 ^a^	23.26 ± 3.20 ^a^	132.46 ± 0.58 ^b^	133.91 ± 0.46 ^a^	723.10 ± 12.7 ^a^

Chemical conditioning (CC). ^a, b^: Different letters in the same column indicate significant differences (*p* < 0.05).

## References

[1] Alimentarius C. (2015). Codex Standard for Named Vegetable Oils. Codex-Stan.

[2] Lai O.-M., Jacoby J.J., Leong W.-F., Lai W.-T. (2019). Nutritional Studies of Rice Bran Oil. Rice Bran Rice Bran Oil.

[3] Sampaio K.A., Zyaykina N., Wozniak B., Tsukamoto J., De Greyt W., Stevens C.V. (2015). Enzymatic Degumming: Degumming Efficiency versus Yield Increase. Eur. J. Lipid Sci. Technol..

[4] Jiang X., Chang M., Jin Q., Wang X. (2015). Application of Phospholipase A1 and Phospholipase C in the Degumming Process of Different Kinds of Crude Oils. Process Biochem..

[5] Cowan D., Korsholm N. Development in Enzymatic Degumming. Proceedings of the Presentation at the 7th Euro Fed Lipid Congress.

[6] Manjula S., Jose A., Divakar S., Subramanian R. (2011). Degumming Rice Bran Oil Using Phospholipase-A1. Eur. J. Lipid Sci. Technol..

[7] Clausen K. (2001). Enzymatic Oil-Degumming by a Novel Microbial Phospholipase. Eur. J. Lipid Sci. Technol..

[8] Sampaio K.A., Zyaykina N., Uitterhaegen E., De Greyt W., Verhé R. (2019). LWT—Food Science and Technology Enzymatic Degumming of Corn Oil Using Phospholipase C from a Selected Strain of Pichia Pastoris. LWT Food Sci. Technol..

[9] Gupta M.K. (2017). Practical Guide to Vegetable Oil Processing.

[10] Nikolaeva T., Rietkerk T., Sein A., Dalgliesh R., Bouwman W.G., Velichko E., Tian B., Van As H., van Duynhoven J. (2020). Impact of Water Degumming and Enzymatic Degumming on Gum Mesostructure Formation in Crude Soybean Oil. Food Chem..

[11] Passos R., Ferreira R., Batrista E., Meirelles A.J.A., Maximo G.J., Ferreira M.C., Sampaio K.A. (2019). Degumming Alternatives for Edible Oils and Biodiesel Production. Food Public Health.

[12] American Oil Chemists’ Society AOCS (2012). Official Method Ca 5a-40, Free Fatty Acids Official Methods and Recommended Pratices of the AOCS.

[13] American Oil Chemists’ Society AOCS (2012). Official method Ce 1-62, acid composition by Gas Chromatography official Methods and recommended Practices of the AOCS. Champaign.

[14] American Oil Chemists’ Society AOCS (2012). Official Method Ca 20-99, Inductively Coupled Plasma (ICP) Official Methods and Recommended Prectices of the AOCS.

[15] American Oil Chemists’ Society AOCS (2012). Official Method Cd 11b-91, Dyglycerides by Capillary Gas Chromatography Official Methods and Recommended Practices of the AOCS.

[16] Diehl B.W.K. (2008). NMR Spectroscopy of Natural Substances. NMR Spectrosc. Pharm. Anal..

[17] Ansolin M., Souza P.T., Meirelles A.J.A., Batista E.A.C. (2017). Tocopherols and Tocotrienols: An Adapted Methodology by UHPLC/MS without Sample Pretreatment Steps. Food Anal. Methods.

[18] Lüdtke F.L. (2016). Estudo da degomagem e clarificação de óleo bruto do farelo de arroz ( Oryza Sativa ). Visando Refino Físico.

[19] Qureshi A.A., Khan D.A., Mahjabeen W., Qureshi N. (2015). Dose-Dependent Modulation of Lipid Parameters, Cytokines and RNA by [Delta]-Tocotrienol in Hypercholesterolemic Subjects Restricted to AHA Step-1 Diet. Br. J. Med. Med. Res..

[20] Van Hoed V., Depaemelaere G., Ayala J.V., Santiwattana P., Verhé R., De Greyt W. (2006). Influence of Chemical Refining on the Major and Minor Components of Rice Bran Oil. J. Am. Oil Chem. Soc..

[21] Wang O., Liu J., Cheng Q., Guo X., Wang Y., Zhao L., Zhou F., Ji B. (2015). Effects of Ferulic Acid and –Oryzanol on High-Fat and High-Fructose Diet-Induced Metabolic Syndrome in Rats. PLoS ONE.

[22] Dijkstra A.J. (2009). Recent Developments in Edible Oil Processing. Eur. J. Lipid Sci. Technol..

[23] Lamas D.L., Constenla D.T., Raab D. (2016). Effect of Degumming Process on Physicochemical Properties of Sunflower Oil. Biocatal. Agric. Biotechnol..

[24] Jiang X., Chang M., Wang X., Jin Q., Wang X. (2014). A Comparative Study of Phospholipase A 1 and Phospholipase C on Soybean Oil Degumming. J. Am. Oil Chem. Soc..

[25] De Greyt W. (2012). Current and Future Technologies for the Sustainable and Cost-Efficient Production of High Quality Food Oils. Eur. J. Lipid Sci. Technol..

[26] Yang B., Zhou R., Yang J.G., Wang Y.H., Wang W.F. (2008). Insight into the Enzymatic Degumming Process of Soybean Oil. J. Am. Oil Chem. Soc..

[27] Jahani M., Alizadeh M., Pirozifard M., Qudsevali A. (2008). Optimization of Enzymatic Degumming Process for Rice Bran Oil Using Response Surface Methodology. LWT Food Sci. Technol..

[28] Dayton C.L.G., Galhardo F. (2015). Enzymatic Degumming Utilizing a Mixture of PLA and PLC Phospholipases. U.S. Patent.

[29] Van hoed V. (2010). Quality Assessment of the High Value Vegetable Oils by Characterization of Minor Components. Fac. Biosci. Eng. Ghent Univ. Belgium..

[30] Krishna A.G.G., Khatoon S., Shiela P.M., Sarmandal C.V., Indira T.N., Mishra A. (2001). Effect or Reffining of Crude Rice Bran Oil on the Retention of Oryzanol in the Refined Oil. J. Am. Oil Chem. Soc..

